# Signal Expansion Method in Indoor FMCW Radar Systems for Improving Range Resolution

**DOI:** 10.3390/s21124226

**Published:** 2021-06-20

**Authors:** Seongmin Baek, Yunho Jung, Seongjoo Lee

**Affiliations:** 1 Department of Information and Communication Engineering & Convergence Engineering for Intelligent Drone, Sejong University, Seoul 05006, Korea; seongmin@itsoc.sejong.ac.kr; 2 Department of Smart Drone Convergence, School of Electronics and Information Engineering, Aerospace University, Goyang-si 10540, Korea; yjung@kau.ac.kr

**Keywords:** FMCW, FMCW radar, range resolution, signal extension

## Abstract

As various unmanned autonomous driving technologies such as autonomous vehicles and autonomous driving drones are being developed, research on FMCW radar, a sensor related to these technologies, is actively being conducted. The range resolution, which is a parameter for accurately detecting an object in the FMCW radar system, depends on the modulation bandwidth. Expensive radars have a large modulation bandwidth, use the band above 77 GHz, and are mainly used as in-vehicle radar sensors. However, these high-performance radars have the disadvantage of being expensive and burdensome for use in areas that require precise sensors, such as indoor environment motion detection and autonomous drones. In this paper, the range resolution is improved beyond the limited modulation bandwidth by extending the beat frequency signal in the time domain through the proposed Adaptive Mirror Padding and Phase Correction Padding. The proposed algorithm has similar performance in the existing Zero Padding, Mirror Padding, and Range RMSE, but improved results were confirmed through the $\rho_{s}$ indicating the size of the side lobe compared to the main lobe and the accurate detection rate of the OS CFAR. In the case of $\rho_{s}$, it was confirmed that with single targets, Adaptive Mirror Padding was improved by about 3 times and Phase Correct Padding was improved by about 6 times compared to the existing algorithm. The results of the OS CFAR were divided into single targets and multiple targets to confirm the performance. In single targets, Adaptive Mirror Padding improved by about 10% and Phase Correct Padding by about 20% compared to the existing algorithm. In multiple targets, Phase Correct Padding improved by about 20% compared to the existing algorithm. The proposed algorithm was verified through the MATLAB Tool and the actual FMCW radar. As the results were similar in the two experimental environments, it was verified that the algorithm works in real radar as well.

## 1. Introduction

Compared to other sensors (camera, LiDAR, etc.), the radar has relatively few malfunctions in various weather conditions or external environments, ensuring the reliability of the system [1,2]. For this reason, the radar is used in the autonomous driving industry for unmanned objects such as drones and automobiles [3,4,5,6,7,8,9,10]. In addition, the characteristics of a radar are such that the antenna and transmitter/receiver can be miniaturized and quantified, and they operate with low power consumption [11,12]. The advantage of such a radar is a sensor suitable for IoT (Internet of Things) applications such as bio-signal detection and gesture recognition [13,14].

The FMCW (Frequency Modulated Continuous Wave) radar, one of several radar types, refers to a radar that continuously radiates a frequency modulated signal. Unlike other types of radars, the FMCW radar has the advantage of being able to detect multiple targets. For this reason, the FMCW radar is used as a suitable sensor to detect indoor objects [15,16,17,18,19,20,21].

**Figure 1:**
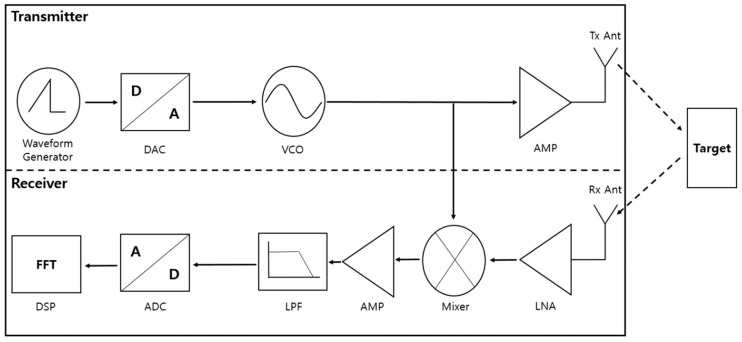
FMCW radar system architecture.

Figure 1 is the system architecture of the transmitter and receiver in a FMCW radar [22]. Figure 1 shows a block diagram of the process in which the signal transmitted from the transmitter hits the target and the reflected signal is received by the receiver. The FMCW radar transmission signal generates a linear transmission waveform through the Waveform Generator and modulates the frequency of the transmission signal through the VCO (voltage control oscillator). After that, the transmission signal is amplified through the amplifier, and then transmitted to the target through the Tx antenna. The time-delayed signal reflected by hitting the target is received through the Rx antenna and passed through a low-noise amplifier (LNA) to obtain a signal of the same frequency as the transmitted signal. The FMCW radar system measures distance and velocity via the beat frequency, which is the difference between the frequencies of the transmitted and received signals. Therefore, in order to obtain the beat frequency, which is the difference between both signals, it is multiplied by a mixer and then passed through an LPF (low pass filter). An analog signal that has passed through LPF into a digital signal through an ADC (analog to digital converter) is then converted. In the DSP (digital signal process) stage, the beat frequency is extracted from the digital signal through FFT (Fast Fourier Transform), and through this, the relative distance, speed, and the angle of the target are calculated.

(1) $$R_{res}=\frac{C}{2BW}$$

Range resolution is a parameter that allows the FMCW radar to accurately measure objects. The formula for calculating the distance resolution is as shown in Equation (1), and its parameters are as follows. In Equation (1), $R_{res}$ is the range resolution, C is the speed of light, and BW is the modulation bandwidth of the FMCW radar. It can be confirmed that the range resolution depends on the specific modulation bandwidth of the FMCW radar [23].

In order to detect an indoor environment with an FMCW radar, it is necessary to use an FMCW radar with a large modulation bandwidth with a resolution as much as a camera. However, the FMCW radar, which has a large modulation bandwidth, is not suitable for use as an indoor environment detection sensor because it is expensive. Currently, relatively inexpensive PIR (Passive IR) sensors and camera sensors are used as indoor environment detection sensors [24]. However, since the PIR sensor simply detects motion according to temperature changes, it cannot detect fine motion, and the camera sensor is also easily affected by light and external environmental factors. Therefore, the use of the radar, which is relatively less sensitive to environmental factors, has emerged. Recently, research is being conducted in the direction of improving the range resolution in a limited modulation bandwidth without increasing the modulation bandwidth of a low-cost FMCW radar with a small modulation bandwidth. One of the several algorithms involves extending the beat frequency signal in the time domain.

Zero and mirror are used as simple signal extension methods used in communication and image processing [25,26]. When this algorithm is applied in FMCW radar DSP, it is as follows. Zero Padding is a method to improve the estimation accuracy by adding a sample having a magnitude of 0 after the signal when a signal containing a beat frequency enters the DSP stage of the Digital signal. Mirror Padding is a method to improve the estimation accuracy by copying a signal containing the beat frequency, turning it over, and attaching it to the end of the signal. However, when FFT is performed to estimate the distance between these two algorithms, side lobes other than the Main Lobe are generated, and the ability to distinguish between objects deteriorates.

Another range resolution enhancement algorithm is Signal Resampling [27]. It is an algorithm that improves the range resolution by re-sampling the signal at the target’s main lobe point. The algorithm uses Phase Correlation to increase the complexity of the algorithm by using FFT and IFFT in the process of resampling the signal. Also, it is difficult to apply the algorithm to multiple targets. For this reason, it is not suitable for improving the range resolution of low-cost FMCW radars through uncomplicated algorithms in indoor environments.

Recently, an algorithm was developed that expands the signal through Deep Learning [28]. It learns a signal containing the beat frequency, which is an original signal through deep learning, and expands the front and back of the signal to improve the distance resolution. Although the range resolution has been improved through the algorithm, there is a disadvantage in applying it in an indoor environment. First, a network design for learning is required, and complexity increases by extracting a signal of a desired band through Zoom FFT. Also, as a problem caused by generating a new signal through deep learning, if the object to be measured is close, the information on the target disappears. When detecting an object with an object detection sensor in an indoor environment, the resulting object disappearance can be fatal. Therefore, it is not appropriate to use in an indoor environment because it cannot afford the risk of such complexity and the disappearance of information on attached objects.

In this paper, two algorithms are proposed to reduce the side lobe that occurs while improving the range resolution. The first algorithm proposed is Adaptive Mirror Padding, which improves the distance estimation accuracy by extending the signal acquired in the time domain without discontinuity of magnitude. The proposed second algorithm is Phase Correct Padding, which is a method of improving the distance estimation accuracy by extending the signal acquired in the time domain through the FMCW radar without a phase error.

The order of the contents of this paper is as follows. Section 2 explains the basic principles of FMCW radar and how to estimate range. Section 3 describes an algorithm for improving range resolution through Adaptive Mirror Padding and Phase Correct Padding algorithms. In Section 4, we verify the results of the simulation through MATLAB and the algorithm through the actual FMCW radar. Finally, conclusions are drawn in Section 5.

## 2. Overview of the FMCW Radar and Range Measuring

Waveform types of FMCW radar are sawtooth and triangular waves. This section explains the overview and range measuring through sawtooth waveforms.

**Figure 2:**
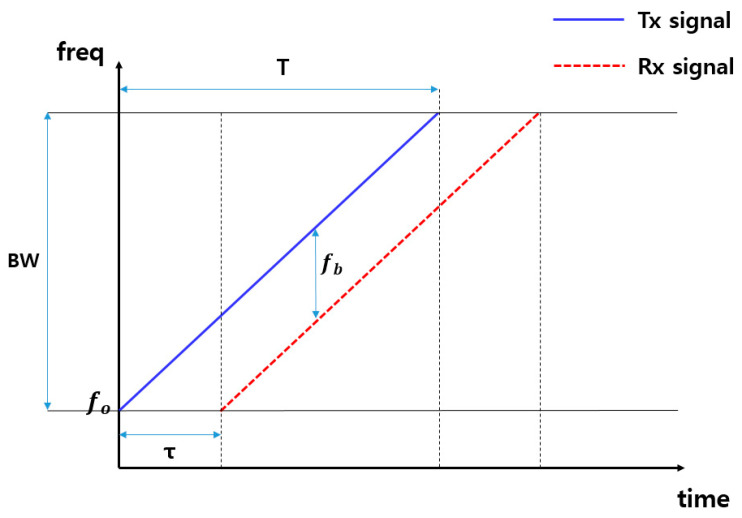
Transmit and receive frequency signals over time in FMCW radar.

Figure 2 shows the FMCW radar sawtooth wave transmission and reception signals over time. In this graph, the blue line is the transmitted signal, and the red line shows the signal received by reflecting the stopped object. The transmitted signal is reflected and received by the object to be measured, and the frequency difference appears in proportion to the delay time (τ).

(2) $$r=\frac{\tau \cdot C}{2}$$

Equation (2) expresses the distance between the radar and the target to be measured by using the delay time (*τ*). In the above equation, τ is the delay time, *C* is the speed of light, and *r* is the distance between the radar and the target to be measured.

(3) $$f_{ideal}=\frac{2\cdot BW\cdot r}{C\cdot T_{m}}$$

Equation (3) expresses the beat frequency using the distance. This makes it possible to obtain an ideal beat frequency corresponding to the distance between the radar and the target. In the above equation, BW is the modulation bandwidth, *r* is the distance, *C* is the speed of light, $T_{m}\;$is Sweep time, $\mathrm{and}\;f_{ideal}$ is the ideal beat frequency.

(4) $$FFT_{bin}=\frac{F_{s}}{N_{fft}}$$

Equation (4) express the FFT bin spacing. The number of FFT points is $N_{fft}$, and $F_{s}$ is the sampling rate. $FFT_{bin}$ is the minimum interval at which a signal can be represented in the frequency domain.

**Figure 3:**
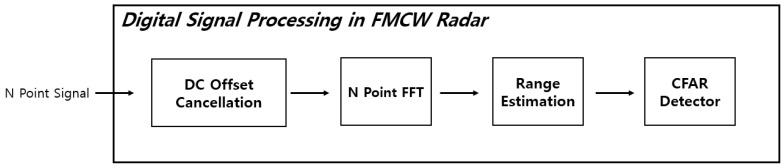
FMCW radar DSP architecture.

Figure 3 shows a structure in which a signal sample of N point digitally converted through ADC at the receiving end operates in DSP. First, the DC value of the signal is removed, and the beat frequency is extracted by taking an N point FFT.

If the target measured by the FMCW radar is a single target, one Max Value is generated among the N Point FFT results, and the corresponding index is extracted.

(5) $$i_{max}=arg_{max}\left|X\left(i\right)\right|$$

In Equation (5), X(*i*) means the output value at the *i*-th bin after performing the FFT.

(6) $$R_{est}=R_{res}\cdot i_{max}$$

If the bit frequency corresponding to the range resolution and the minimum ${\mathrm{FFT}}_{bin}$ is matched one-to-one, the distance can be estimated through the FFT Bin Point Index as shown in Equation (6). Through this, range estimation of the target to be measured is performed.

The CFAR (Constant False Alarm Rate) algorithm is used to check whether the target is detected [29,30,31]. The CFAR algorithm is detects a target to be detected during radar signal processing. The CFAR algorithm uses a method of variably adjusting the threshold to have a fixed false alarm rate by setting a fixed false alarm rate, measuring fluctuating noise as an average clutter power, and multiplying the coefficient corresponding to the false alarm rate. The threshold value and the result of the FFT are compared, and if the threshold is large, it is determined as a target, and if it is small, it is determined as not a target. According to the method of observing the average clutter power, there are CA (Cell Averaging) and OS (Order Statistic).

## 3. Proposed Algorithm

**Figure 4:**
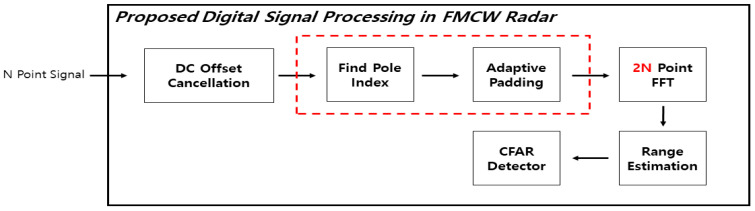
Proposed FMCW radar DSP architecture.

In this paper, the range resolution is improved through two algorithms. The first method is Adaptive Mirror Padding. The main point of this algorithm copies the signal based on the poles. The direction of copying changes according to the number of data lost during the copying process, and the method of padding is also determined. In this section, it is assumed that the padding is done only once for the purpose of explaining the algorithm. The method of the algorithm will be explained with reference to Figure 4 below.

**Figure 5:**
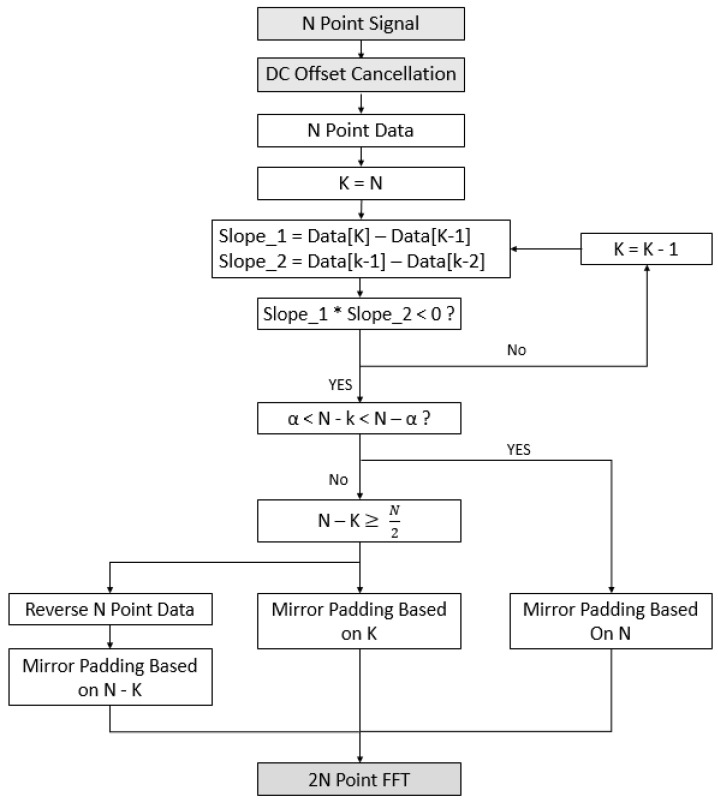
Proposed Adaptive Mirror Padding algorithm flowchart.

**Figure 6:**
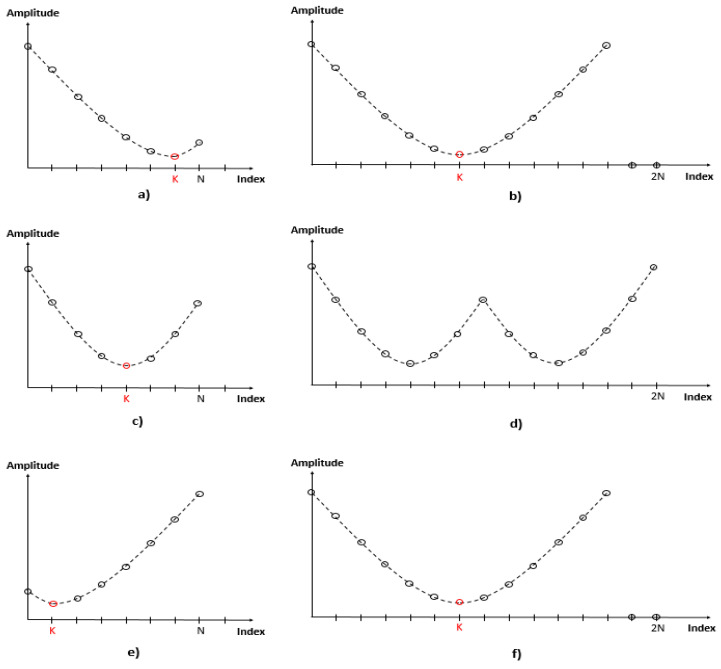
Adaptive Mirror Padding according to the index position of the pole.

Figure 4 shows the Architecture of the FMCW radar DSP with the proposed algorithm added. Figure 5 is a flow chart for the algorithm, and Figure 6 explains the algorithm’s process.

In Figure 5, the signal data sampled at N points is received from the DSP after passing through the ADC. First, the number of samples of the data is stored as K, and an algorithm to find the pole is performed. In the method of finding the pole, two slopes Slope1 and Slope2 are obtained through three samples from the last sample (K) of the received signal. At this time, if the two slopes are positive, it is determined that they are not poles, and in order to compare the next three samples, K = K − 1 and then the next point is compared. If the two slopes are different, the position is determined as the pole and proceeds to the next. After finding the position of the pole, whether to perform Mirror Padding or Pole Mirror Padding is determined according to the number of lost samples. The optimal value of the α point was made through an experiment to find the optimal alpha point in the Simulation Section.

Figure 6 shows an algorithm that is padded to Adaptive based on the position of the index of the pole. First, (a), (c) and (e) will be described separately based on the position of Index K. In the case of (a), the number of samples lost is N-K. If N-k is smaller than the set number of samples, mirror padding is performed on the pole based on Index K, and padding is performed as shown in (b). In the case of (c), if the number of lost samples N-K is greater than the set number of samples, mirror padding is performed without applying the pole algorithm, and padding is performed as shown in (d). In the case of (e), if the index K of the pole is the front part of the signal, the signal is inverted. After that, the number of lost samples is compared, and if it is less than the set value, pole mirror padding is performed, and if it is greater than the set value, mirror padding is performed. (f) shows the case in which the number of lost samples is greater than the set value.

After performing Adaptive Mirror Padding, a 2 N point FFT is performed by padding zeros by the number of lost samples to minimize data loss.

The second algorithm is Phase Correct Padding. The main point of the algorithm is copied after correcting the phase of the signal in order to maintain the linearity of the phase in the process of copying and pasting the original signal. The contents of the algorithm will be explained through the following figure.

**Figure 7:**
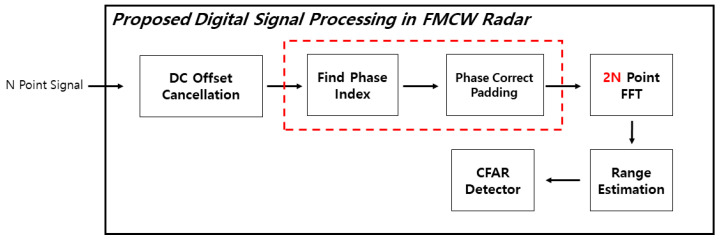
Proposed FMCW radar DSP Architecture.

**Figure 8:**
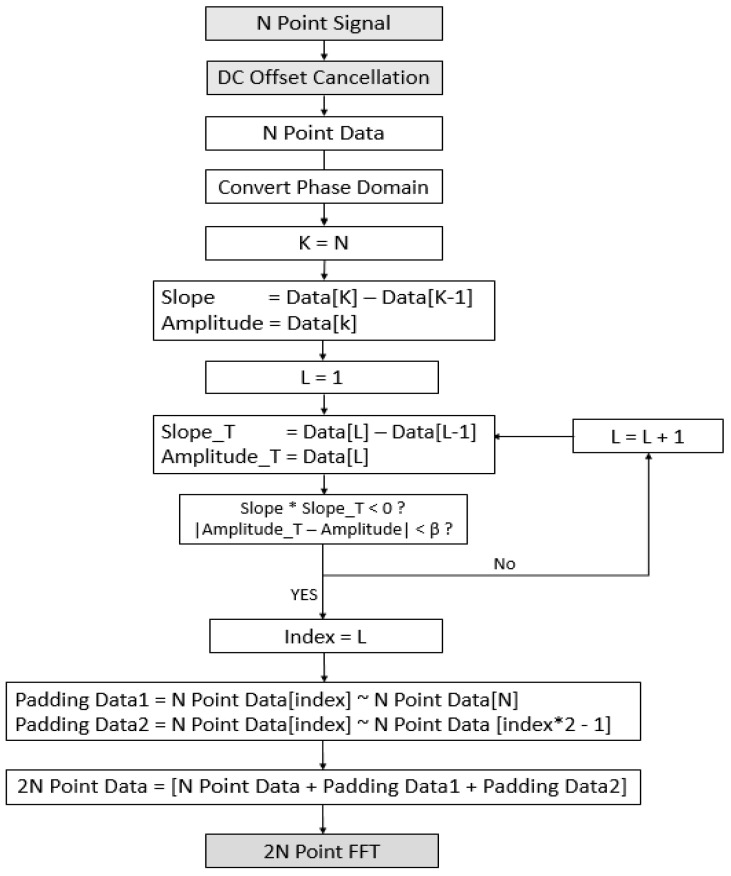
Proposed Phase Correct algorithm flowchart.

**Figure 9:**
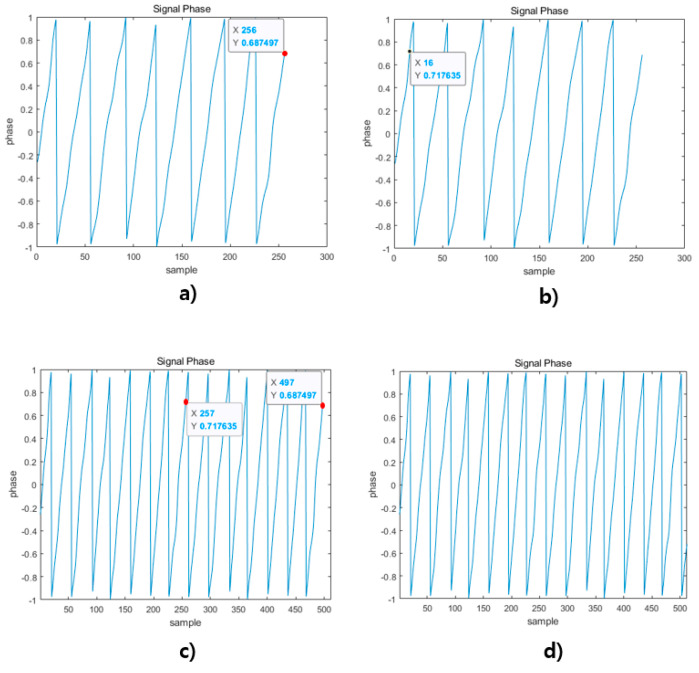
Process of obtaining the Phase Correct Padding algorithm index.

Figure 7 shows the addition of the Phase Correct Padding algorithm to the existing DSP architecture. Figure 8 is a flow chart for the algorithm, and Figure 9 is a figure explaining the process of the algorithm.

Figure 8 describes the flow chart of the algorithm. To start the algorithm, an N point digital signal containing the beat frequency is received from the FMCW radar. The received signal is applied to the algorithm after removing the DC Offset. N Point Digital Signal to which DC offset is applied is called N Point Data. To apply this algorithm, we need to know the phase and magnitude of each sample. Therefore, the algorithm starts by changing to the phase domain. The first step of the algorithm is to find the phase slope and magnitude of the last sample of N point data. After that, the sample at the point most similar to the slope and magnitude of the last sample obtained earlier is compared from the first index of N Point Data to obtain it. The optimal value of the threshold β set for comparison of similar points was found through experiments in the Section 4. The L Index the point where the magnitude and phase of the last sample are closest. For Padding, set the following two Padding Data. Padding Data 1 refers to samples from L index to N Index of N Point Data and Padding Data 2 refers to samples from L index to 2 × L − 1 index of N Point Data. 2 N Point Data is an extension signal in which Padding Data1 and Padding Data2 are sequentially padded after the original signal, N Point Data. After that, 2 N Point FFT is performed to extract the distance. If the input signal is non-uniformly sampled, the distance can be extracted using NUFFT [32,33].

Figure 9 shows the application process of the algorithm: (a) is the process of obtaining the slope and magnitude of the signal of the last sample in the Convert Phase Domain, (b) is the process of finding the sample of the L Index mentioned above, (c) shows the padding process of Padding Data1 behind the original signal, and (d) shows the signal extended to the final 2 N Point Sample by padding to Padding Data2.

To verify the algorithm proposed in this paper, a $\rho_{s}\;$representing the ratio of the side lobe to the main lobe was defined. The definition of $\rho_{s}$ is as follows:

(7) $$k=arg_{max}(E_{i}),\;0\leq i<N$$

(8) $$\tilde{E_{n}}=\frac{E_{n}}{\sum_{i=0}^{N-1}E_{i}}$$

(9) $$\rho_{s}=\frac{\sum_{n=0,\;n\neq k}^{N-1}\tilde{E_{n}}}{\tilde{E_{k}}}$$

In Equation (7), k is the Index of Bin with Maximum Energy. In Equation (8), $E_{i}\;$means the energy of the *i*-th bin, and $\tilde{E_{n}}$ means the *n*-th normalized energy.

**Figure 10:**
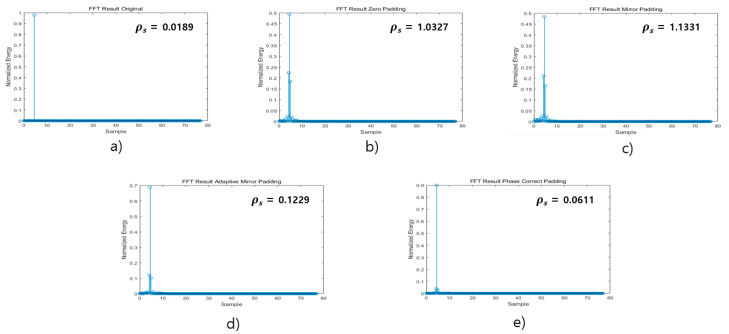
FFT results by algorithm.

Figure 10 is the result of Single Target FFT for each algorithm. The setting parameters are as follows. In the case of (a), the modulation bandwidth is 500 MHz, and the range resolution is 0.3 m according to Equation (1). In the case of (b)–(e), the modulation bandwidth is 250 MHz, and the range resolution is 0.6 m by Equation (1). In case (b) to (e), the distance estimation accuracy is improved by applying each algorithm to a signal with a modulation bandwidth of 250 MHz. TX_SNR is 10 db, and assuming that the target distance is 4.5 m, the results (a) to (e) were all obtained at the correct FFT bin location. As a result of estimating the ratio of the main lobe to the side lobe through the $\rho_{s}$ in Equation (8), the proposed Phase Correct Padding was confirmed to be 0.0611, which was the most similar to (a), and then the Adaptive Mirror Padding was 0.1229. In the case of the conventional padding, Zero Padding and Mirror Padding, the results were 1.0327 and 1.1331, respectively. Through this, it was confirmed that the proposed algorithm significantly reduces the occurrence of side lobes, and when the CFAR algorithm is applied to detect the target later, it is possible to obtain the advantage of being able to accurately detect the target.

## 4. Simulation

In this paper, we verified the performance of the proposed algorithm using MATLAB Tool and FMCW radar.

### 4.1. MATLAB Simulation

**Figure 11:**
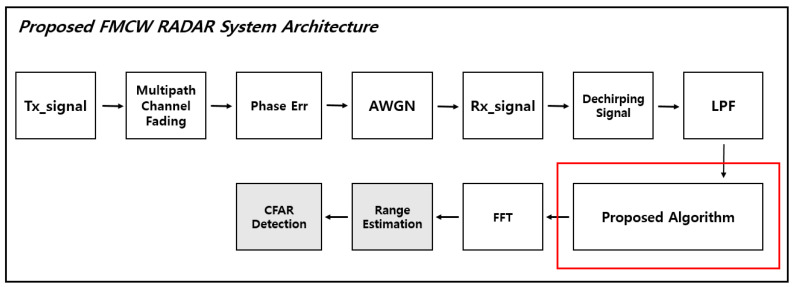
Algorithm simulation flowchart.

Figure 11 is a block diagram of a simulation through MATLAB. In order to implement the simulation environment as close as possible to the real environment, the following environment was added, and radar parameters such as the actual radar to be used are shown in Table 1. The added simulation environment is as follows. First, the center frequency of the FMCW radar used in the indoor environment is 24 GHz, so a 24 GHz indoor environment multipath fading was added [34]. Phase Error was added ±20 ppm at the center frequency, and AWGN (Additive White Gaussian Noise) was added.

MAE (Mean Absolute Error) is a value obtained by converting the difference between an actual value and a predicted value into an absolute value and taking the average. In this paper, it is defined as the following Equation (10):

(10) $$\mathrm{MAE}=\frac{\sum_{i=1}^{N}\left|\tilde{y_{i}}-\tilde{x_{i}}\right|}{N}$$

In Equation (10), $\tilde{y_{i}}\;$is the *i*-th value of the normalized FFT result of the signal to which the algorithm is not applied, and $\tilde{x_{i}}$ is the *i*-th value of the normalized FFT result of the signal to which the algorithm is applied. N is the total number of samples.

**Figure 12:**
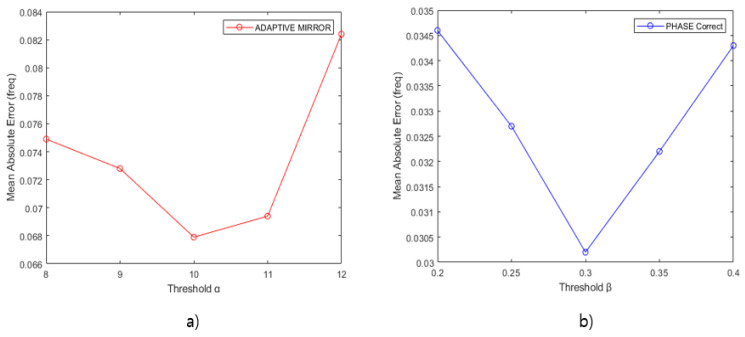
Optimal Threshold Value α, β.

Figure 12 shows the MAE results according to α and β to obtain the optimal α and β values in the proposed algorithms, Adaptive Mirror Padding and Phase Correct Algorithm. (a) is the MAE result according to alpha in Adaptive Mirror Padding, and (b) is the MAE result according to beta in the Phase Correct Algorithm. The simulation environment was conducted in the FMCW radar environment as shown in Table 1 with Tx_SNR of 20 db. The number of repetitions is 1, and MAE measurement was performed in a single target. In the MAE result of the Phase Correct Algorithm, it can be confirmed that the MAE result is the best when the value of β is 0.3. Therefore, α and β of the algorithm were set to 10 and 0.3, respectively, and simulation was performed.

**Figure 13:**
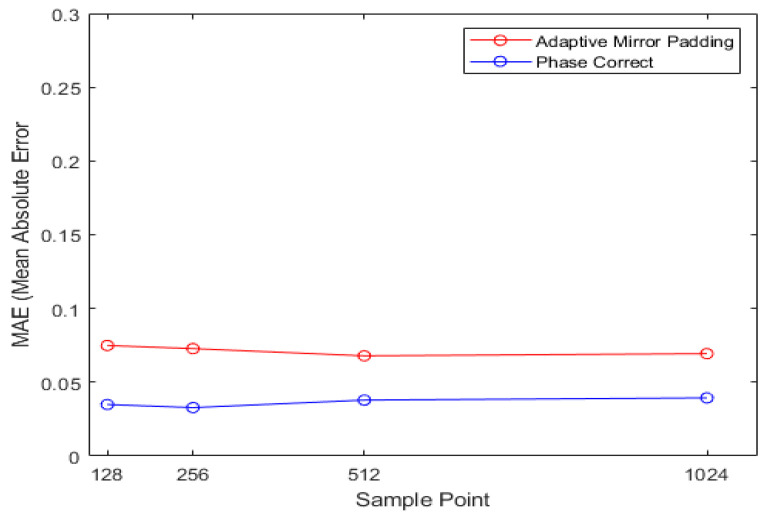
MAE result of the proposed algorithm according to Sample Point.

Figure 13 shows the MAE results of the proposed algorithm for each sample point. In Table 1, by adjusting the sampling frequency and sweep time, the sample point was changed to 128, 256, 512, and 1024 to confirm the MAE result. The simulation environment in Figure 13 is the same as the simulation environment in Figure 12. The proposed algorithm extends the signal with beat frequency information in the time domain. For example, if the input signal is 128 points and the number of iterations of the algorithm is 1, the FFT is taken by extending the signal to 256 points. From the graph above, it can be seen that the MAE results for each sample point are all similar. Later, the performance of the algorithm is evaluated using the actual FMCW radar, and the sample point of the actual FMCW radar is fixed at 256 points. Therefore, in order to proceed in the same way as in the real environment, the point of the sample was fixed at 256 and the simulation was performed.

The performance verification method of the proposed algorithm and comparison algorithm was verified through MAE, Range RMSE (Root Mean Square Error), OS-CFAR (Ordered Statistic-Constant False Alarm Rate), and $\rho_{s}$. The limit of the number of repetitions of the proposed algorithm was measured through MAE, and the performance of each algorithm was verified through Range RMSE, OS-CFAR, and $\rho_{s}$.

#### 4.1.1. Single Target

**Figure 14:**
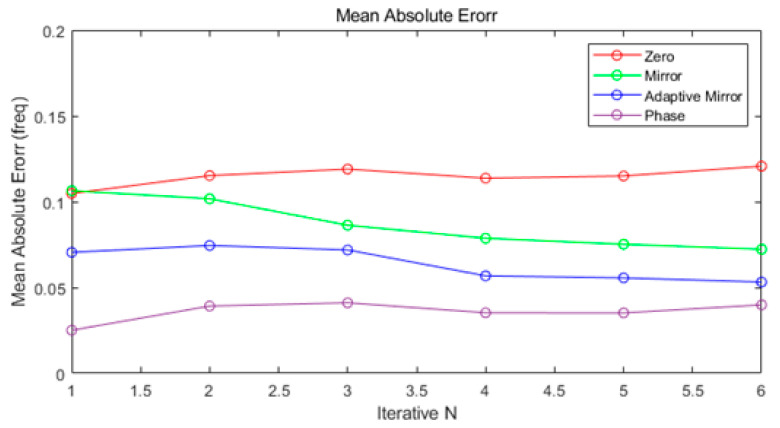
Single Target Mean Absolute Error (Normalized Spectrum).

Figure 14 shows the MAE of each algorithm for Single Target according to the number of repetitions *N*. The experimental environment was conducted in the FMCW radar environment as shown in Table 1, with Tx_SNR between 2 db and 20 db. The comparison algorithm was set to Zero Padding and Mirror Padding. If the number of repetitions *N* = 1, that is, the original signal is padded once through each algorithm, the result of MAE is the best. It can be seen that as the number of iterations increases, the value of MAE no longer increases. Therefore, through the results of MAE in Figure 14, the limit of the number of iterations is defined as 4, the result of MAE is the best when the number is 1, and the results of future simulations are confirmed by setting the number of iterations 1.

(11) $$\mathrm{Range}\;\mathrm{RMSE}=\sqrt{\frac{1}{M}\overset{M-1}{\underset{n=0}{\sum }}{\left(r-r_{est}\right)}^{2}}$$

Equation (11) calculates the Range RMSE in FMCW radar. *M* is the number of FFT points, *r* is the distance of a given target, and $r_{est}$ is the distance of the target estimated by the proposed algorithm.

Table 2 shows the results of Range RMSE and $\rho_{s}$ for Single Target. The environment of the simulation is the same as the experiment environment in Figure 14, and the distance interval between 3 m and 12 m for the target to be measured is set to 0.3 m. The Range estimation was estimated through Equations (5) and (6), and $\rho_{s}$ was obtained through equation 10, and Range RMSE was obtained through equation 11. The difference between the range RMSE between the existing algorithm and the proposed algorithm was insignificant. Compared with the existing algorithm through the result of Low S, which represents the ratio of the main lobe to the side lobe, the adaptive mirror padding is confirmed to be improved by 3 times, while the phase correct padding is improved by 6 times.

Table 3 is the result of the application of OS CFAR to Single Target. The simulation environment is the same as the environment for calculating Range RMSE in Table 2. As mentioned in Section 2, the effects of side lobes other than the main lobe. The parameters of OS-CFAR are as follows: False Alarm Rate = 1e−5, Training Cell = 32, Guard Cell = 2, Rank: 24. The result of OS-CFAR was defined as Accurate Detection Rate as follows. When FFT was taken for a single target, it was defined as a case in which the target was detected at the point where the distance of the given single target and the FFT bin location match. In the case of Zero Padding and Mirror Padding, as a result of applying OS CFAR, it can be seen that the Accurate Detection Rate is lowered due to the side lobe to 74.05% and 65.85%. On the other hand, Adaptive Mirror Padding and Phase Correct Padding are 78.15% and 90.25%, respectively, showing improved performance compared to the zone algorithm. In particular, in the case of Phase Correct Padding, it is improved by about 25% compared to Mirror Padding.

#### 4.1.2. Multiple Target

**Figure 15:**
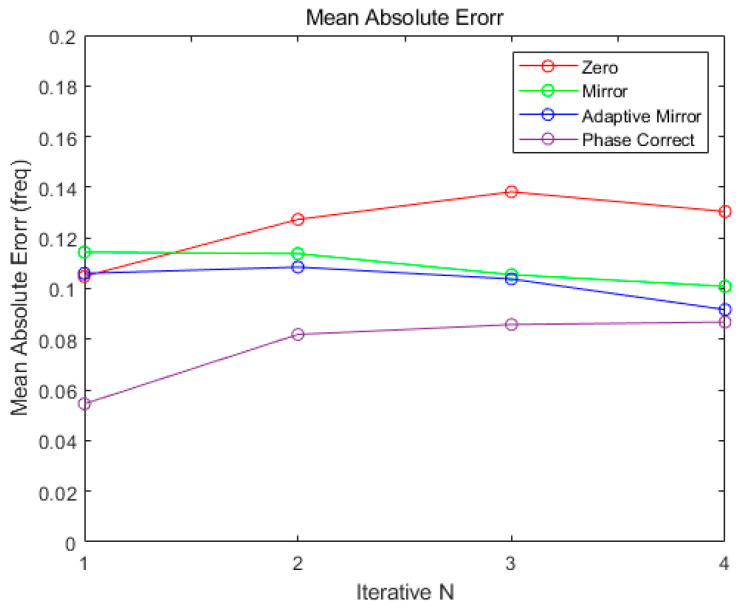
Multiple Target Mean Absolute Error (Normalized Spectrum).

Figure 15 shows the MAE of each algorithm for multiple targets according to the number of iterations. In the same simulation environment as in Figure 14, the number of targets was set from single to multiple (maximum 4). The MAE shows the best for all of algorithms when the number of iterations is 1 as in Single Target Simulation. Therefore, similarly to Single Target Simulation, the simulation result will be verified with the number of iteration being set by 1.

Table 4 shows the results of applying OS CFAR to multiple targets. It was performed according to the same parameters and simulation environment as when the exact detection error rate was extracted by applying OS-CFAR to a single target, and only the number of detection targets was changed from single to multiple. It can be seen that the results of the proposed algorithm are superior to the comparison algorithms, Zero Padding and Mirror Padding, and in the case of Phase Correct Padding, performance is improved by about 13% compared to Zero Padding and by about 20% compared to Mirror Padding.

### 4.2. Test

**Figure 16:**
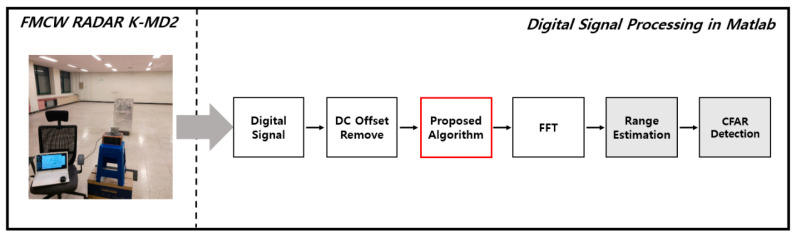
Algorithm Test Flowchart.

Figure 16 shows the environment in which the algorithm was tested using FMCW radar K-MD2, and Table 5 is the specification of the used FMCW radar [35]. It measures data on a single target using FMCW radar K-MD2 and performs signal processing through MATLAB. After removing DC Offset from Digital Signal received from K-MD2, separate I and Q channel data. The algorithm was applied in the same way as in MATLAB simulation.

#### 4.2.1. Single Target

Table 6 shows the results of the Range RMSE and average Low S for Single Target data obtained through K-MD2, an actual FMCW radar. The specifications of the radar are shown in Table 5. The distance between the targets was 1.8 m~3.9 m, and the targets were measured at 0.3 m intervals. Results similar to Table 2, simulated for Single Target with MATLAB, are shown. Compared with the existing algorithm, it can be seen that Adaptive Mirror Padding is improved about 4-fold and Phase Correct Padding is improved by 8 times.

Table 7 shows the results of applying OS-CFAR to single target data obtained through FMCW radar in the same manner as in Table 3. It can be seen that the results of the proposed algorithms, Adaptive Mirror Padding and Phase Correct Padding, improved about 11% and 28% compared to Zero Padding, and about 3% and 20% compared to Mirror Padding. 

#### 4.2.2. Multiple Target

Table 8 shows the application results of OS CFAR for multiple targets. It was conducted in the same measurement environment as the previous experiment involving Single Target. The distance between the targets was 1.8 m to 5.4 m, with the targets at intervals of 0.3 m, and the number of targets was set from single to multiple (up to 4) and the experiment was performed. The results of the proposed algorithm were superior to those of the comparison algorithms, Zero Padding and Mirror Padding, and in the case of Phase Correct Padding, performance was improved by about 11% compared to Zero Padding and by about 16% compared to Mirror Padding.

## 5. Conclusions

In this paper, in order to improve the range resolution, we proposed the Adaptive Mirror Padding and Phase Correct Padding algorithms in order to eliminate discontinuities caused by extending the bit frequency signal in the time domain. The algorithm was verified through the MATLAB Tool and the actual FMCW radar. Performance verification was carried out through the accurate detection rate through the $\rho_{s}$, which indicates the ratio of the main lobe to the side lobe, and the OS CFAR through OS-CFAR. In the result of $\rho_{s}$, in the case of Adaptive Mirror Padding, the single target improved about 3 times compared to the existing algorithm, and the phase correct padding improved about 6 times. In the Accurate Detection result through OS-CFAR, in the case of Adaptive Mirror Padding, about 10% improved compared to the existing algorithm in Single Target, and about 20% in Phase Correct Padding. In multiple targets, it can be seen that the result of the Accurate Detection Rate through OS CFAR is improved by about 20% in Phase Correct Padding compared to the existing algorithm. The proposed algorithm not only solves the reliability problem and complexity by applying it to low-cost IoT FMCW radar sensor used in an indoor environment, but it can also be applied to the SAR (Synthetic Aperture Radar) system [36].

## Figures and Tables

**Table 1:** MATLAB Simulation environment.

Center Frequency	24 GHz
Chirping Bandwidth	250 MHz
Range Resolution	0.6 m
Maximum Distance	20 m
Sampling Frequency	38.46 MHz
Sweep Time	7.2 μs
Sample point	256

**Table 2:** Single Target Range RMSE, $\rho_{s}$.

Algorithm	Average Range RMSE	$\mathrm{Average}\;\rho_{s}$
Zero Padding	0.0611 m	1.1895
Mirror Padding	0.1233 m	1.2384
Adaptive Mirror Padding	0.1476 m	0.4185
Phase Correct Padding	0.0913 m	0.2035

**Table 3:** Single Target OS CFAR result.

Algorithm	Accurate Detection Rate
Zero Padding	74.05%
Mirror Padding	65.85%
Adaptive Mirror Padding	78.15%
Phase Correct Padding	90.25%

**Table 4:** Multiple Target OS CFAR result.

Algorithm	Accurate Detection Rate
Zero Padding	70.34%
Mirror Padding	63.85%
Adaptive Mirror Padding	70.84%
Phase Correct Padding	83.98%

**Table 5:** FMCW radar Spec.

Center Frequency	24 GHz
Chirping Bandwidth	250 MHz
Range Resolution	0.6 m
Maximum Distance	20 m
Sampling Frequency	38.46 MHz
Sweep Time	7.2 μs
Sample point	256

**Table 6:** Single Target Range RMSE, $\rho_{s}$.

Algorithm	Average Range RMSE	$\mathrm{Average}\;\rho_{s}$
Zero Padding	0.0812 m	1.0906
Mirror Padding	0.1628 m	1.1533
Adaptive Mirror Padding	0.1413 m	0.3044
Phase Correct Padding	0.1025 m	0.1158

**Table 7:** Single Target OS CFAR result.

Algorithm	Accurate Detection Rate
Zero Padding	57.74%
Mirror Padding	65.29%
Adaptive Mirror Padding	68.46%
Phase Correct Padding	85.67%

**Table 8:** Multiple Target OS CFAR result.

Algorithm	Accurate Detection Rate
Zero Padding	71.53%
Mirror Padding	66.52%
Adaptive Mirror Padding	72.42%
Phase Correct Padding	82.30%

## Data Availability

Not applicable.
